# Apolipoproteins in Health and Disease

**DOI:** 10.3390/ijms25137048

**Published:** 2024-06-27

**Authors:** Jordi Ordóñez-Llanos, Joan Carles Escolà-Gil

**Affiliations:** 1 Foundation for Clinical Biochemistry & Molecular Pathology, 08041 Barcelona, Spain; 2 Institut de Recerca Sant Pau, 08041 Barcelona, Spain; 3 CIBER de Diabetes y Enfermedades Metabólicas Asociadas (CIBERDEM), 28029 Madrid, Spain

Although apolipoproteins (apo) were initially acknowledged as major determinants in lipoprotein metabolism and cardiovascular disease, the findings of recent studies have revealed the significance of multiple apolipoprotein classes and subclasses in various biological processes and pathophysiological pathways. These include diabetes, cancer, obesity, neurodegenerative disorders, and inflammatory and infectious diseases. We are delighted to provide an overview of the papers included in the present Special Issue, entitled “Apolipoproteins in Health and Disease”, which was produced with the aim of publishing apt and informative findings on the molecular aspects of apolipoproteins and their impact on health and disease risk.

Population-based registries are helpful tools for monitoring diseases or their associated factors and deriving conclusions for their management. The Epidemiological Study on the Prevalence of Arterial Hypertension and Cardiovascular Risk in Romania (SEPHAR) is a nationwide registry surveilling the prevalence of arterial hypertension and its associated cardiovascular disease (CVD) risk [1]. The investigators involved in the SEPHAR study published a substudy of the registry [2]. The substudy describes the evaluation of apolipoprotein B (apoB) concentration, among other CVD risk factors, and its relationship with the occurrence of subclinical atherosclerosis in a population of ~800 Romanian adult subjects aged < 80 years. Local registries on CVD and their risk factors are of great interest not only to the global health community but also to local health authorities in particular since the prevalence of CVD risk factors in different countries can be quite variable, as are the actions needed for their control. For example, in the Dorobantu study [2], active smoking was recorded in ~85% of participants, and these data outline the need to focus not only on arterial hypertension to prevent CVD but smoking-cessation campaigns. The study authors subdivided the participants into two subgroups by apoB concentration using a cut-off value of 130 mg/dL (1.30 g/L). Subjects with higher apoB concentrations were less likely to be smokers, though the comparison was between 77% vs. 86% smokers, and older; no differences were found in CVD antecedents or risk factors. Not surprisingly, subjects with higher apoB concentrations showed a worse lipid profile. When the subjects were grouped into four apoB categories from <65 mg/dL to >100 mg/dL, a significant trend toward higher fasting glucose and urate concentrations and %HbA1c was observed. Lastly, significant carotid plaques detected using ultrasonography were also more frequent in subjects with apoB concentrations >130 mg/dL. Most of the results concurred with the concept reviewed in the Special Issue by Nelson et al. [3] in that apoB concentration measures more accurately the number of atherogenic “inputs” interacting with arterial walls than any of the so-called “atherogenic cholesterols”, i.e., low-density lipoprotein (LDL), very-low-density lipoprotein (VLDL), and non-high-density lipoprotein (HDL) cholesterol. Thus, a clinically relevant suggestion to be taken from the SEPHAR study is that apoB concentration should be considered when evaluating the atherosclerotic risk associated with lipids.

The article by Nelson et al. [3] reviews two main points: first, cholesteryl ester transfer protein (CETP) inhibition or modulation as a pharmacological target for dyslipidemia therapy and, second, the usefulness of apoB concentration as a biomarker for evaluating the effectiveness of CETP inhibition and, importantly, other dyslipidemia therapies. CETP activity decreases HDL cholesterol (HDLc) concentration and increases LDL cholesterol (LDLc) concentration by promoting the transfer of cholesteryl esters from HDL particles to triglyceride-rich particles and that of triglycerides to HDL in a reverse sense. Inhibition of CETP will result, among other features, in an increase in HDLc that in turn is nearly always associated with a decrease in atherosclerotic CVD. The first developed CETP inhibitors improved subjects’ lipid profile but failed to significantly decrease the risk of atherosclerotic CVD and one of them even increased it [4]. As a result, these drugs are not in clinical use. However, this current picture may change if the evaluation of obicetrapib, a new CETP inhibitor currently being tested in a phase III trial, provides positive results. In a previous phase II trial, obicetrapib at a dose of 10 mg/d was associated with a significant increase in HDLc concentration (up to 170%) and a reduction in that of LDLc by up to 44%, lipoprotein(a) by up to 56.5%, and apolipoprotein B by up to 27% [5]. The greater effect of obicetrapib on lipids compared to previous CETP-inhibiting drugs was attributable to its higher binding affinity for CETP, among other chemical features [5,6]. The effect of obicetrapib on apoB is a remarkable feature since it is known that not only LDLc and non-HDLc concentration but also apoB concentration are causative factors of atherosclerotic CVD. Although it has been stated in the guidelines of the European Society of Cardiology that information on CVD risk provided by apolipoprotein B is similar to that provided by LDLc and non-HDLc [7], a very recent study by Sniderman, a co-author of the currently referenced review, showed that for the same LDLc, non-HDLc or triglyceride CVD risk was increased or decreased according to the increased or decreased apoB concentration [8]. In summary, the review provides strong evidence that apoB is a more accurate marker of CVD risk than LDLc, non-HDLc, or triglycerides and, as a consequence, any therapy improving apoB levels such as obicetrapib will result in a net benefit in decreasing atherosclerotic CVD risk.

The relationship between *APOE* gene polymorphisms and CVD, particularly coronary heart disease (CHD), was first reported in the 1980s. The authors of earlier studies observed that carriers of the apoE4 phenotype were more frequent among patients with myocardial infarction than carriers of apoE3 (wild type) or apoE2 phenotypes [9], suggesting that lipids were the link between the phenotypes and CHD [10]. However, meta-analyses developed thereafter found heterogeneity among those previous and subsequent results. One of these meta-analyses described an increased risk of CHD up to 42% higher in apoE4 carriers than in apoE3 carriers [11]. However, in comparison with other meta-analyses describing a close-to-linear relationship between apoE phenotypes and LDLc concentrations and CHD risk, only a slightly higher (6%) risk in apoE4 carriers was reported compared with apoE3 carriers; in comparison, apoE2 carriers showed a lower risk [12]. These differences in CHD risk associated with apoE phenotypes suggest that external factors, not reviewed in these studies, could explain the discrepancy in the results. Kofler et al. [13] analyzed the interaction between apoE genotypes and body mass index (BMI), gender, and inflammatory biomarkers associated with CVD. They concluded that stratification by sex and adiposity should be considered when analyzing the association between apoE phenotypes and CHD risk. Ozen et al. [14] further extended the results of Kofler in the BODYCON study presented in the current Special Issue. The BODYCON study involved 360 healthy women and men, in which refined anthropometric measures such as fat and lean body mass, visceral fat, android and gynoid fat, and their ratios were evaluated and their dietary intake was recorded. The study provided interesting results beyond what was previously known. To explore the interaction between body mass and apoE phenotypes, anthropometry, CVD biomarkers, and diet, the participants were divided by BMI. Notably, the main differences in lipid profile according to apoE phenotypes were restricted to apoE4 participants with BMI < 25.0 kg/m^2^, who, in turn, had significantly higher lean and android body mass. Moreover, a different dietary pattern consisting of higher energy and fiber intake and lower protein and trans-fat intake was also found in apoE4 carriers. The above results, if confirmed in other studies, indicate that body fat distribution and diet potentially determine players involved in the interplay between apoE phenotypes, lipid profile, and CVD.

The introduction of next-generation sequencing has greatly advanced gene analysis, reducing both the time required and costs involved per sample. This technology has facilitated the discovery of numerous *APOB* variants, thereby enhancing the genetic diagnosis of familial hypercholesterolemia (FH) caused by mutations in this gene [15]. The study by Rodríguez-Jiménez et al. [16] aimed to identify APOB variants in 825 index patients with clinically suspected FH and conduct segregation and functional analyses to determine their pathogenicity. Overall, 40% of the patients had variants in low-density lipoprotein receptor (*LDLR*), *APOB*, proprotein convertase subtilisin/kexin type 9, or LDLR adaptor protein 1, with 12% of these variants found in *APOB*. These variants were present in less than 0.5% of the general population and were classified as damaging or probably damaging by three or more pathogenicity predictors. The study focused on characterizing the c.10030A>G; p.(Lys3344Glu) and c.11401T>A; p.(Ser3801Thr) variants. The p.(Lys3344Glu) variant co-segregated with high levels of LDL cholesterol in the two families studied. LDL isolated from patients heterozygous for the apoB p.(Lys3344Glu) variant showed a reduced ability to compete with fluorescently labeled LDL for cellular binding and uptake compared to control LDL, and it was significantly less effective in supporting cell proliferation. Conversely, LDL carrying the apoB p.(Ser3801Thr) variant was not defective in competing with control LDL for cellular binding and uptake. Overall, the p.(Lys3344Glu) variant was identified as a causative factor for hypercholesterolemia, whereas the p.(Ser3801Thr) variant, previously classified as being of uncertain significance, was found to be benign.

Abnormal apo levels are linked to metabolic syndrome development [17]. Numerous studies have demonstrated that acute lymphoblastic leukemia (ALL) survivors frequently experience the early onset of various metabolic disorders, including obesity, dyslipidemia, hypertension, and metabolic syndrome (MetS) [18]. A key criterion of metabolic syndrome—visceral obesity observed in ALL survivors—results in several dysfunctions, particularly pertaining to lipid metabolism. Sztolsztener et al. [19] conducted a cross-sectional study to investigate treatment-related changes in apo levels as potential markers for obesity in ALL survivors and examined the relationships between weight, gender, anticancer treatment, and apo concentrations. The results of the study revealed that childhood ALL survivors with a history of anticancer treatment are at high risk of developing altered apo profiles. Specifically, changes were observed in apoA1, apoA2, and apoD levels compared to healthy individuals. Additionally, a significant correlation was found between increased BMI and elevated apoC1 concentration in these patients. The results of this study suggest that specific apolipoproteins could serve as biomarkers for metabolic imbalance, providing valuable prognostic information for the development of overweight status and obesity in ALL survivors. The findings indicate that abnormal apo composition may contribute to the onset of MetS, including significant weight gain, hypertriglyceridemia, and hypercholesterolemia. Therefore, systematic follow-up for MetS is essential in this population. The study underscores the importance of long-term monitoring for metabolic complications directly associated with anticancer treatment in children with hematological malignancies, highlighting the need for early intervention and management strategies to address these potential risks.

The molecular mechanisms involved in the binding of HDL to cells, resulting in particle or lipid uptake and/or signaling, are poorly understood, partly because the inventory of HDL receptors appears to be incomplete [20]. Frei et al. [21] aimed to better understand the interplay between cell surface receptors and HDL functionality by characterizing the HDL receptome. They used chemoproteomic technologies, including automated cell surface capturing (auto-CSC) and HATRIC-based ligand–receptor capturing (LRC), to identify and quantify N-linked glycosylated receptors at the cellular surface. The auto-CSC technology identifies the acute N-glycosylated surfaceome, while HATRIC-LRC identifies unknown receptors for known ligands such as HDL using a trifunctional cross-linker strategy. To create a cell surface atlas of potential HDL-interacting proteins, the authors applied auto-CSC to model systems mimicking tissues relevant for reverse cholesterol transport and commonly used in HDL research. They focused on tissue-specific receptor neighborhoods that act as hubs for translating extracellular signals into cellular responses. These signaling hubs are dynamic and can be influenced by external stimuli, affecting ligand–receptor interactions. The authors studied these receptor neighborhoods by treating human endothelial cells with vascular endothelial growth factor A, which is known to mediate the translocation of scavenger receptor B1 to the cell surface, leading to significant changes in the HDL–receptor interaction landscape. Using HATRIC-LRC, the authors identified endothelial HDL receptors and discovered tyrosine-protein kinase Mer, a receptor involved in maintaining vascular cell homeostasis, as a modulator of HDL binding and uptake. Their findings demonstrate that the HDL receptome is complex and dynamic, with receptor nanoscale organization potentially influencing HDL binding and uptake.

ApoA1, the major protein component of HDL, has been implicated in diseases such as atherosclerosis and amyloidosis, where insoluble aggregates are deposited in organs and arteries [22]. These aggregates often take the form of amyloid fibrils, suggesting that amyloid formation may contribute to disease progression [23]. Frankel et al. [24] examined the aggregation of both wild-type apoA1 and a mutant form, apoA1 K107D, under various conditions. They characterized the aggregates based on size, morphology, and protein conformation using optical spectroscopy, dynamic light scattering, and cryo-electron microscopy. The study authors found that apoA1 can form two main types of aggregates: globular condensates, which maintain a helical conformation, and sheet-rich amyloid fibrils. The formation of amyloid fibrils, which are rich in beta-sheets, was promoted by oxidation or mutation. The K107D mutant in particular exhibited a higher tendency to form amyloid fibrils. The end state of aggregation for this mutant often included a mixture of beta-sheet-rich fibrils and alpha-helix-rich condensates. The above findings suggest that the aggregation of apoA1 into different structures may vary depending on the disease and the affected body part, indicating a complex relationship between apoA1 aggregation and disease pathology.

Acute administration of apoA4 protein has been found to increase thermogenesis and fatty acid uptake in the brown adipose tissue (BAT) of mice [25]. This finding indicates that apoA4’s peripheral effects may stimulate BAT thermogenesis and influence lipid metabolism. Larussa et al. [26] investigated the impact of sustained endogenous apoA4 levels on BAT thermogenesis and obesity resistance in mice. The study involved transgenic mice with increased apoA4 production (APOA4-Tg) and wild-type mice subjected to either a standard chow diet or a high-fat diet (HFD) for 4 or 10 weeks. Key parameters assessed included body weight gain, feeding behavior, BAT thermogenic and lipolytic proteins, and plasma lipid levels. The results of the study indicated that maintaining elevated apoA4 levels in APOA4-Tg mice led to significant enhancements in UCP1-dependent BAT thermogenesis and overall energy expenditure. This thermogenic activation helped mitigate the typical body weight and fat mass increases associated with HFD consumption. Additionally, APOA4-Tg mice exhibited lower plasma lipid concentrations, further demonstrating a protective effect against HFD-induced obesity. The study authors concluded that high endogenous apoA4 levels promote BAT thermogenesis and energy expenditure, thereby reducing weight gain, fat accumulation, and plasma lipids in response to HFD. The above findings suggest a potential therapeutic role for apoA4 in preventing diet-induced obesity.

As referenced above, apoE deficiency and polymorphisms are linked to numerous chronic diseases, including atherosclerosis, obesity/diabetes, and Alzheimer’s disease. The human *APOE* gene is expressed in various cell types such as hepatocytes, adipocytes, immune cells, vascular smooth muscle cells, and brain cells. Alagarsamy et al. [27] reviewed the role of apoE in cardiovascular, metabolic, and neurological health, highlighting how apoE deletion and polymorphisms contribute to disease pathogenesis. Key features of apoE’s role in health and disease include its function as a ligand transport protein, signaling molecule via cell surface receptors and proteoglycans, modulator of cell homeostasis, and regulator of the inflammatory response. Mechanistic insights from these studies could optimize treatment strategies for individuals at high risk of cardiometabolic and neurological diseases due to *APOE* gene polymorphisms. Proposed and tested approaches in preclinical models include the use of apoE mimetic peptides, apoE4 structural correctors, modulators of apoE trafficking pathways, apoE antisense oligonucleotides, and *APOE* gene therapy. The challenge remains to specifically target these treatments to appropriate tissues and cell types to maximize effectiveness and minimize adverse effects. In this context, Borràs et al. [28] reviewed the mechanisms of HDL-mediated cholesterol trafficking in the central nervous system (CNS) and its implications for Alzheimer’s disease (AD). The review authors highlight the transportation of cholesterol via HDL in the CNS and its relationship with amyloid-beta peptide accumulation. ApoE is a key structural component of HDL-like lipoproteins in the CNS, playing a crucial role in their metabolism. Evidence suggests decreased cholesterol efflux to cerebrospinal fluid (CSF) HDL-like lipoproteins in AD patients; however, studies have largely been conducted in cells not relevant to CNS pathology [28]. The potential of AD patients’ CSF to induce cholesterol efflux from human astrocytes has not been thoroughly investigated in well-defined AD patient cohorts, representing a significant gap in research. HDL-like-mediated cholesterol uptake studies indicate that cholesterol entry into neurons may be decreased in AD, possibly contributing to impaired amyloid-beta clearance and tau protein accumulation. Overall, the review authors call for more research to clarify the role of HDL-like cholesterol trafficking in the brain and its impact on AD pathology.

The cholesterol metabolite 27-hydroxycholesterol accumulates in papillary thyroid carcinoma (PTC) cells, promoting their aggressive behavior [29]. Furthermore, increased expression of LDLR and enhanced LDL uptake in various tumors has been linked to cancer growth [30]. Revilla et al. [31] examined the role of LDL in enhancing the aggressiveness of PTC via the mitogen-activated protein kinase (MAPK) signaling pathway. The authors studied the effect of LDL in two thyroid cell lines: TPC1 (wild type) and BCPAP (BRAFV600E mutant). The results of this study indicate that the presence of LDL can accelerate oncogenic processes through the MAPK pathway, particularly in the presence of the BRAFV600E mutation. This synergy between LDL-mediated receptor uptake and BRAFV600E suggests a potential for worse clinical outcomes in hypercholesterolemic patients with PTC. The results of the study imply that LDLR is significant in the MAPK signaling cascade in thyroid cancer. Consequently, the authors propose that cholesterol-lowering drugs, in combination with MAPK inhibitors, might serve as an effective therapeutic strategy for treating aggressive PTC.

In conclusion, we hope that the above articles, contributed by experts in the field, will provide valuable resources to researchers studying apolipoproteins and their influence on various diseases.

## Abbreviations

ALL	Acute lymphoblastic leukemia
AD	Alzheimer’s disease
Apo	Apolipoprotein
APOA4-Tg	Transgenic mice with increased apoA4 production
Auto-CSC	Automated cell surface capturing
BAT	Brown adipose tissue
CETP	Cholesteryl ester transfer protein
CHD	Coronary heart disease
CVD	Cardiovascular disease
CNS	Central nervous system
CSF	Cerebrospinal fluid
FH	Familial hypercholesterolemia
HDL	High-density lipoprotein
HDLc	High-density lipoprotein cholesterol
HFD	High-fat diet
LDL	Low-density lipoprotein
LDLc	Low-density lipoprotein cholesterol
LDLR	LDL receptor
LRC	Ligand–receptor capturing
MAPK	Mitogen-activated protein kinase
MetS	Metabolic syndrome
PTC	Papillary thyroid cancer
SEPHAR	Study on the Prevalence of Arterial Hypertension and Cardiovascular Risk
